# Issues associated with the use of phosphospecific antibodies to localise active and inactive pools of GSK-3 in cells

**DOI:** 10.1186/1745-6150-6-4

**Published:** 2011-01-24

**Authors:** Victor M Campa, Robert M Kypta

**Affiliations:** 1 Cell Biology and Stem Cells Unit, Center for Cooperative Research in Biosciences (CIC bioGUNE), Bizkaia Technology Park, 48160 Derio, Spain; 2 Department of Surgery and Cancer, Imperial College London, London W12 0NN, UK

## Abstract

**Abstract:**

**Background:**

Glycogen synthase kinase-3 (GSK-3) is a ubiquitously expressed serine/threonine (Ser/Thr) kinase comprising two isoforms, GSK-3α and GSK-3β. Both enzymes are similarly inactivated by serine phosphorylation (GSK-3α at Ser21 and GSK-3β at Ser9) and activated by tyrosine phosphorylation (GSK-3α at Tyr279 and GSK-3β at Tyr216). Antibodies raised to phosphopeptides containing the sequences around these phosphorylation sites are frequently used to provide an indication of the activation state of GSK-3 in cell and tissue extracts. These antibodies have further been used to determine the subcellular localisation of active and inactive forms of GSK-3, and the results of those studies support roles for GSK-3 phosphorylation in diverse cellular processes. However, the specificity of these antibodies in immunocytochemistry has not been addressed in any detail.

**Results**

Taking advantage of gene silencing technology, we examined the specificity of several commercially available anti-phosphorylated GSK-3 antibodies. We show that antibodies raised to peptides containing the phosphorylated Ser21/9 epitope crossreact with unidentified antigens that are highly expressed by mitotic cells and that mainly localise to spindle poles. In addition, two antibodies raised to peptides containing the phosphorylated Tyr279/216 epitope recognise an unidentified protein at focal contacts, and a third antibody recognises a protein found in Ki-67-positive cell nuclei. While the phosphorylated Ser9/21 GSK-3 antibodies also recognise other proteins whose levels increase in mitotic cells in western blots, the phosphorylated Tyr279/216 antibodies appear to be specific in western blotting. However, we cannot rule out the posssibility that they recognise very large or very small proteins that might not be detected using a standard western blotting approach.

**Conclusions**

Our findings indicate that care should be taken when examining the subcellular localisation of active or inactive GSK-3 and, furthermore, suggest that the role of GSK-3 phosphorylation in some cellular processes be reassessed.

**Reviewers**

Dr. David Kaplan, Dr. Robert Murphy and Dr. Cara Gottardi (nominated by Dr Avinash Bhandoola.)

## Background

Glycogen synthase kinase-3 (GSK-3) is a multifunctional serine/threonine (Ser/Thr) kinase first identified by its ability to phosphorylate and inactivate glycogen synthase. Since then, more than fifty substrates have been identified and GSK-3 has been found to be involved in multiple cellular functions including protein synthesis, microtubule organization, cell migration, cell proliferation, apoptosis and differentiation [1-3]. There are two isoforms of GSK-3, GSK-3α and GSK-3β, and there are two splicing variants of the latter, β1 and the brain-specific isoform, β2, which appears to play a unique role in axon growth [4]. GSK-3α and GSK-3β are 98% identical within their kinase domains but they are not functionally identical, since GSK-3β mutant mice die during embryonic development [5,6]. In resting cells, GSK-3 is active, being phosphorylated at a tyrosine (Tyr) residue in the activation loop (Tyr279 in GSK-3α and Tyr216 in GSK-3β) [7]. Cell stimulation by several growth factors activates Akt/PKB, which phosphorylates a serine residue close to the amino terminus (Ser21 in GSK-3α and Ser9 in GSK-3β) to inhibit kinase activity [8,9]. Other extracellular signals also lead to changes in GSK-3 localisation or activity, for example, activated G proteins induce relocalisation and activation of GSK-3 at the membrane [10] and inducers of stress and/or apoptosis induce GSK-3 tyrosine phosphorylation and nuclear localisation [11].

GSK-3 activity can be directly assayed *in vitro *using kinase assays either in immune precipitates or directly from extracts [12]. However, these methods are time consuming and, in practice, GSK-3 activity is frequently indirectly inferred by western blotting to determine its phosphorylation state or the phosphorylation state of known substrates. In addition, immunocytochemistry using phosphospecific antibodies has been used to determine the subcellular localisation of active or inactive forms of GSK-3 [13-16]. The correlation between GSK-3 phosphorylation and kinase activity is well established and therefore these approaches are widely used [17]. The antibodies are raised to short peptides corresponding to phosphorylated sites in GSK-3 and are normally validated by incubation with the peptide immunogen, pre-treatment of samples with phosphatase, or by observing an increase in signal upon stimulation with factors known to modulate GSK-3 activity, insulin for Ser9/21 phosphorylation, for example. Although a loss of signal upon addition of the peptide immunogen or an increase in the signal after insulin treatment is indicative of a functional antibody, it does not exclude recognition of other proteins. Similarly, loss of signal upon incubation with phosphatase only excludes recognition of unphosphorylated proteins. This potential lack of selectivity is less of an issue in western blotting since crossreactivity is often revealed by the apparent molecular mass of the proteins being detected. In contrast, when using procedures, such as immunostaining and flow cytometry, it is crucial to address the issue of selectivity [18-20].

Phosphorylation of GSK-3 at Ser21/9 is mediated by several members of the AGC family of kinases, including Akt/PKB [9,21]. Once phosphorylated, this domain of GSK-3 competes with primed substrates for binding to the catalytic domain [8]. Thus, it is possible that the phosphorylated site shares some structural similarity with primed substrates. In addition, the GSK-3 tyrosine phosphorylation site is within the activation loop of the catalytic domain, which is similar in sequence to the activation loops in other CMGC family kinases. Since the motifs involved in GSK-3 activation and inactivation share structural characteristics with other proteins, antibodies recognizing these motifs might be predicted to recognize those same proteins. Ideally a negative control is required using cells do not express the protein of interest. The fact that GSK-3 is ubiquitously expressed as two isoforms with high sequence homology has made this difficult to do in the past, but it can now be done simply, using gene silencing technology.

Taking advantage of RNA interference methods to specifically silence GSK-3α and/or GSK-3β in 22Rv1 prostate cancer cells, we have examined the specificity of commonly used anti-pSer and anti-pTyr GSK-3 antibodies. We have found by western blotting that the anti-pSer antibodies recognise additional proteins, and by immunofluorescence that both the anti-pSer and the anti-pTyr GSK-3 antibodies show nonspecific staining. In summary, our data validate the use of these antibodies for immunoblotting but suggest that they should be used with care when used for immunocytochemistry.

## Results

### Silencing of GSK-3α and GSK-3β isoforms using specific shRNAs

**Figure 1:**
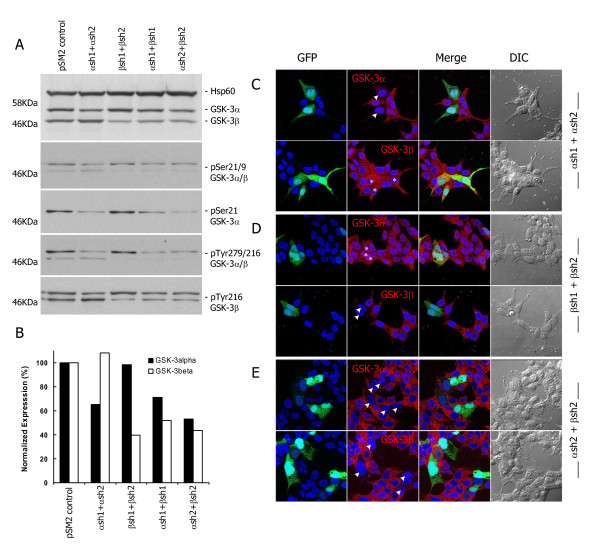
**Silencing of GSK-3 isoforms in 22Rv1 cells**. (A) 22Rv1 cells were transfected with the indicated shRNAs and then selected with puromycin, as described in the Methods. After 72 hours, total cell extracts (20 μg) were prepared and probed by western blotting using the indicated phosphospecific antibodies and then stripped and reprobed with a mixture of antibodies to GSK-3α, GSK-3β and HSP60 (top panel). (B) Expression of GSK-3 normalized to HSP60. (C-E) Cells were co-transfected with a combination of eGFP and the pSM2 plasmids αsh1 and αsh2 (C), βsh1 and βsh2 (D) or αsh2 and βsh2 (E). 72 hours after transfection, cells were fixed and stained with anti-GSK-3α or anti-GSK-3β monoclonal antibodies and analyzed by confocal microscopy. Arrowheads indicate GFP+ cells in which GSK-3α or GSK-3β are silenced and asterisks indicate GFP+ cells with the same level of protein expression as neighbouring cells. Scale bars = 50 μm.

During our studies of the role of GSK-3 in prostate cancer, we observed that antibodies raised to phosphorylated GSK-3 showed contradictory staining patterns, although each of them was consistent with the technical information provided by manufacturers and with previously published reports. For example, in 22Rv1 cells, mouse anti-GSK-3β (pY216) from BD shows bright nuclear staining similar to that on the data sheet, whereas the rabbit anti-GSK-3α/β (pY279/216) from Merck shows staining at focal adhesions, as previously reported [22]. Noting that the sequences surrounding pY279 in GSK-3α and pY216 in GSK-3β are identical, these results suggest either that each antibody recognises a different pool of tyrosine-phosphorylated GSK-3 or that they bind to different proteins, which raises questions about their specificity. To address this, we silenced GSK-3α and/or GSK-3β in 22Rv1 cells by transfecting shRNAs targeting specific GSK-3 isoforms [4]. As expected, immunoblotting showed a reduction in the expression levels of the targeted GSK-3 isoforms when compared to shRNA controls (Figure 1A, B). Similar reductions in the levels of phosphorylated GSK-3 were detected when probing with anti-GSK-3α/β (pS21/9), anti-GSK-3α (pS21), anti-GSK-3α/β (pY279/216) and anti-GSK-3β (pY216) (Figure 1A). These results indicate efficient reduction of GSK-3 levels and validate the use of these antibodies for immunobloting. Silencing of specific GSK-3 isoforms was further confirmed by immunofluorescence. GSK-3α, but not GSK-3β, staining intensity was reduced in cells co-transfected with eGFP plasmid and shRNAs targeting GSK-3α (GFP+) compared to adjacent non-transfected cells (GFP-) (Figure 1C). In addition, GSK-3β but not GSK-3α staining was reduced in cells co-transfected with shRNAs targeting GSK-3β (GFP+) compared to adjacent non-transfected cells (GFP-) (Figure 1D) and, finally, in both cases fluorescence intensity was just above background levels in cells co-transfected with eGFP plasmid (GFP+) and shRNAs targeting each of GSK-3α and GSK-3β, whereas it was high in adjacent non-transfected cells (GFP-) (Figure 1E). In control experiments over 90% of GFP-positive cells co-expressed the shRNA plasmid, as reflected by a reduction in the expression level of the target protein (Additional file 1). It should be noted that not all of the cells that survived the short treatment with puromycin were transfected (we estimate 30%). The extracts are therefore derived from a mixture of transfected and non-transfected cells, whereas the images shown are of transfected cells in which GSK-3 was downregulated. This explains the apparent discrepency between the observed decreases in expression by western analysis and by immunocytochemistry.

### Anti-pSer GSK-3 antibodies recognise unidentified antigens in mitotic cells

**Figure 2:**
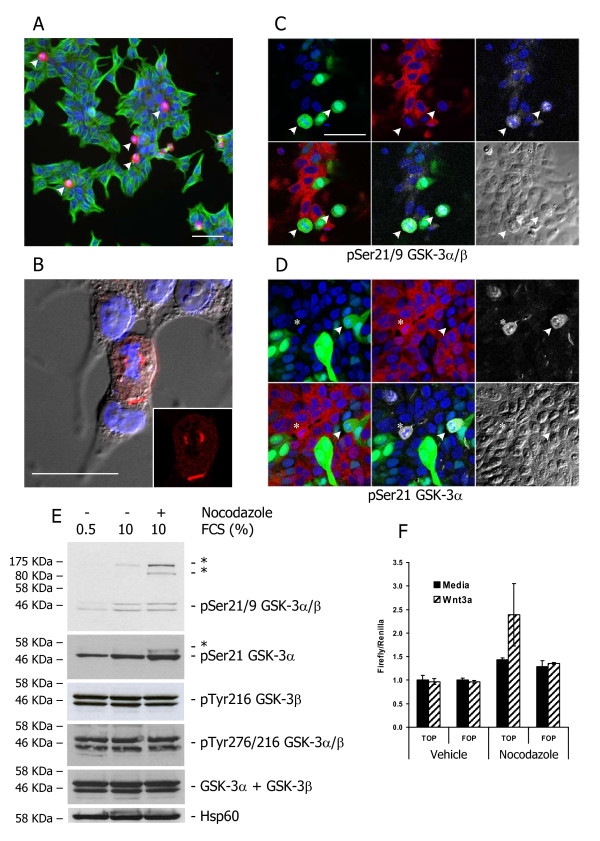
**Anti-GSK-3α/β pS21/9 and anti-GSK-3α pS21 recognise an unidentified antigen in mitotic cells**. (A and B) 22Rv1 cells cultured in normal media growing at 60-70% confluence were fixed and stained with anti-GSK3α/β (pS21/9) (red) and anti-tubulin (green) (A) or with anti-GSK-3α pS21 (red) (B); note the strong staining of mitotic cells by the pS21/9 antibodies (arrowheads). (C and D) Cells were co-transfected with eGFP, αsh2 and βsh2 plasmids and, after 72 h, fixed and stained using a mix of anti-GSK3α and anti-GSK-3β monoclonal antibodies (C and D, red) and anti-GSK-3α/β pS21/9 (C) or anti-GSK-3α pS21 (D) (both far-red, shown in white). Confocal images were obtained as described in the Methods. Arrowheads indicate mitotic transfected (GFP+) cells and asterisks indicate mitotic non-transfected cells (GFP-). In (C) and (D) the bottom right panels show DIC images of the cells. (E) Extracts from cells were cultured in medium containing 0.5% serum, normal medium (10% serum) or normal medium containing nocodazole for 18 h were probed using the indicated antibodies. Asterisks mark nonspecific antigens upregulated in nocodazole treated cells. (F) 22Rv1 cells were transfected with 100 ng TopFlash or FopFlash luciferase reporters and 15 ng pGL3 Renilla luciferase, cultured for an additional 12 h in the absence or presence of nocodazole (300 ng/ml) and then stimulated with recombinant Wnt3a (R&D Systems, 100 ng/ml) for 8 h. Firefly luciferase activity was normalised using *Renilla *luciferase activity. Scale bars: A, C, D = 50 μm; B = 25 μm.

Once we had confirmed the specific silencing of GSK-3 isoforms, we addressed questions about the selectivity of the different anti-phosphorylated GSK-3 antibodies. First, we examined the selectivity of anti-GSK-3α/β (pS21/9) and anti-GSK-3α (pS21) (Cell Signalling Technology). Both antibodies similarly stained cells strongly in all phases of mitosis, with the intensity of the staining highest at the spindle poles (Figure 2A, B), suggesting a possible role for GSK-3 during spindle dynamics, as previously reported by Wakefield et al. [13], who used one of these antibodies. However, when cells were co-transfected with shRNAs targeting both GSK-3α and GSK-3β, there was no difference in the staining intensity in transfected mitotic cells compared with adjacent untransfected cells (Figure 2C). In this figure transfected cells are in green, total GSK-3 in red and the antigen detected by anti-GSK-3α/β (pS21/9) in white. As expected, the level of GSK-3 was reduced in all transfected (GFP+) cells, mitotic and non-mitotic. However, the arrows indicate two GSK-3-negative cells in mitosis that are strongly stained by the GSK-3α/β (pS21/9) antibody. Similar results were obtained using anti-GSK-3α (pS21). In the example shown (Figure 2D), the arrows indicate two cells in mitosis, one that expresses GSK-3 and the other that does not, yet both are recognised by the anti-GSK-3α (pS21) antibody. Thus, the antigen detected by anti-pSer antibodies in mitotic cells is not GSK-3 but presumably another phosphoprotein whose level of expression is increased at mitosis. In fact, western blotting of cell extracts from 22Rv1 cells synchronized at G2/M by treatment with nocodazole revealed several higher molecular weight proteins that were specifically recognised by anti-pSer GSK-3 antibodies, but not by anti-pTyr GSK-3 or total GSK-3 antibodies (Figure 2E). In addition, serum starvation, which is expected to reduce the proportion of cells in mitosis, reduced the signal of the crossreacting antigens. As expected, serum starvation reduced phosphorylation at Ser9/21, most likely through a reduction in Akt/PKB activity, but did not affect GSK-3 phosphorylation at Tyr216/279. Interestingly, although the antigen detected at spindle poles in mitotic cells is not GSK-3, western blots revealed an increase in the pSer21 GSK-3α signal in nocodazole-treated cells, and this correlated with an increase in β-catenin/Tcf transcriptional activity during mitosis (Figure 2F), as previously reported by Davidson et al. [23].

### Anti-pTyr GSK-3 antibodies recognise unidentified antigens in the nucleus and in putative focal adhesions

**Figure 3:**
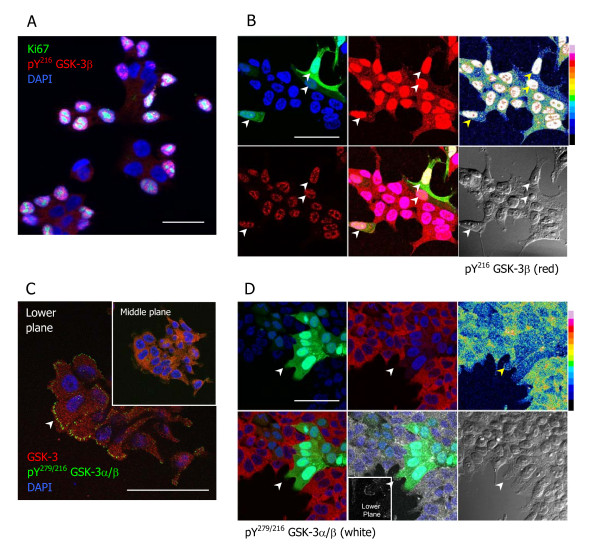
**Anti-GSK-3 pTyr antibodies recognise additional antigens in cell nuclei and at cell borders**. (A) 22Rv1 cells cultured in normal medium until they reached 60-70% confluence were fixed and stained with anti-GSK3β (pY216) antibody (red) and anti-Ki67 (green); (B) Cells were co-transfected with a eGFP, αsh2 and βsh2 plasmids and, after 72 h, fixed and stained using anti-GSK-3β pY216 (red). Transfected cells are shown in green (indicated by arrowheads). Long exposures of the red channel (top and bottom images in middle and top right image) were taken to show that the weak cytoplasmic signal detected by anti-GSK-3β pY216 is reduced in transfected cells (best observed in the top right panel by comparing the signal intensity in the cytoplasm of transfected cells with that in nearby untransfected cells, refer to the adjacent pseudocolor scale). (C and D) Untransfected cells (C) or cells transfected with eGFP, αsh2 and βsh2 plasmids (D) were fixed and stained using a mix of anti-GSK-3α and anti-GSK-3β monoclonal antibodies (red) and anti-GSK-3α/β pY279/216 from Abcam (far red, shown as white in GFP transfected cells). Images were obtained using a confocal microscope as described in the Methods. In (C) the pY279/216 antibody staining of putative focal contacts is clearest in the lower focal plane (arrowhead), whereas total GSK-3 (red) staining is diffuse. The arrowhead in (D) indicates a transfected (GFP+) cell that does not express GSK-3 but is stained at putative focal contacts using the pY279/216 antibody. The inset shows this staining at a focal plane closer to the coverslip. In (B) and (D) the bottom right panels show DIC images of the cells. Scale Bars = 50 μm.

Next, we investigated the selectivity of a mouse monoclonal anti-phosphotyrosine GSK-3β antibody (BD) and two rabbit polyclonal antibodies: anti-GSK3α/β (Y279/216) (Abcam and Merck) and observed quite different staining patterns: the monoclonal antibody showed strong nuclear staining (Figure 3A), whereas both polyclonal antibodies stained what appeared to be focal contacts (Figure 3C, D and Additional file 2); in all cases faint cytosolic staining was also observed. Not all nuclei stained with the anti-GSK-3β (pY216) mouse monoclonal antibody, but those that did were also positive for Ki-67, indicating that they are proliferating cells (Figure 3A). In the case of the anti-GSK3α/β (pY279/216) antibodies, the staining, although variable from cell to cell, was usually localised to one end of each cell (Figure 3C), suggesting that the antigen recognised is expressed by migrating cells. When cells were co-transfected with GFP plasmid and shRNAs targeting both GSK-3α and GSK-3β there was no change in the intensity of the nuclear staining observed using the anti-GSK-3β (pY216) monoclonal antibody (Figure 3B, arrowheads), or in the polarised cytosolic staining observed using the anti-GSK-3α/β (pY279/216) polyclonal antibodies in transfected cells (Figure 3D, arrowhead and Additional file 2) compared to control cells. However, there was a reduction in the intensity of the weak cytosolic staining in both cases (Figure 3B, D; note the pseudocolour scales for relative signal intensities). These data strongly suggest that the antigens recognised in the nucleus and at putative focal contacts by these antibodies are not GSK-3, and that only the weak cytoplasmic staining, where GSK-3α and GSK-3β can be detected using specific antibodies, represent tyrosine-phosphorylated GSK-3.

## Discussion

Several growth factors activate Akt/PKB to inhibit GSK-3 by inducing phosphorylation at Ser21/9 [9] and other external stimuli have been show to increase phosphorylation at Tyr279/216, which is required for GSK-3 activity [7,16,24]. Thus, the phosphorylation state of GSK-3 is widely used to provide an indication of its activity; indeed several companies provide kits to measure GSK-3 activity based on the level of Ser21/9 phosphorylation. Most commercial suppliers sell antibodies raised to short GSK-3-based phosphopeptides that are validated to differing degrees, but include competition with blocking peptide, loss of signal upon treatment with a phosphatase and increased signal after growth factor stimulation. However, these assays do not eliminate the possibility that the antibodies recognise other phosphoproteins.

Using shRNAs to silence GSK-3α, β or both, we checked the selectivity of five different antibodies. Our results indicate that all detected phosphorylated GSK-3 by immunoblotting, with relatively weak binding to other proteins. Anti-GSK-3α/β (pS21/9) and anti-GSK-3α (pS21) recognised nonspecific bands, particularly in extracts from cells treated with nocodazole, but in both cases these could be distinguished from GSK-3 by their molecular masses. In contrast, when the antibodies were used for immunofluorescence, there were three clear instances where they did not perform as expected. First, anti-GSK-3α/β (pS21/9) and anti-GSK-3α (pS21) both associated with unidentified proteins localised at the spindle poles of mitotic cells. This correlated with the appearance of the nonspecific bands in cells treated with nocodazole, suggesting that they recognise phosphoproteins involved in mitosis. Interestingly, the same anti-GSK-3α/β (pS21/9) antibody has been used previously to localise GSK-3 at the spindle poles of mitotic cells and to provide evidence to support a role for GSK-3 inactivation during chromosome alignment [13]. This contrasts with recent reports which indicate that GSK-3β inhibition causes defects in chromosome alignment [25-27], and thus suggesting that active rather than inactive GSK-3 is required for proper spindle formation. Second, the anti-GSK-3β (pY216) monoclonal antibody showed weak cytoplasmic staining and strong nuclear staining in 22Rv1 cells. However, only the weaker staining in the cytoplasm was reduced upon GSK-3 depletion. Active GSK-3 can be found in the nucleus in some cells, where it phosphorylates cyclin D1 and promotes its nuclear export, thus inhibiting proliferation [28]. However, the nuclear staining observed using the GSK-3 (pY216) monoclonal antibody was observed in Ki67-positive cells and did not correlate with the cytoplasmic staining observed using GSK-3α and GSK-3β antibodies. Finally, we observed that the anti-GSK-3 α/β (pY279/216) antibodies from Abcam and Merck recognised what appear to be focal contacts. Again, this signal was not reduced in GSK-3-silenced cells, where we only observed a decrease in the weak cytoplasmic staining. Immunofluorescence studies have been used to show that tyrosine-phosphorylated GSK-3 localises to focal adhesions [14,15]. In contrast, our data suggest that tyrosine-phosphorylated GSK-3 is evenly distributed, similar to GSK-3 itself.

## Conclusions

While our data support the use of phosphospecific GSK-3 antibodies to monitor the phosphorylation state of GSK-3 in cell extracts, they raise doubts about results obtained using these antibodies to study subcellular localisation. The nonspecific recognition of phosphorylated epitopes may prove to be a common issue for antibodies raised to regulatory and target sites of protein kinases that have many substrates.

## Methods

### Cell Culture, plasmids and transfections

22Rv1 cells (ATCC) were cultured in RPMI/DMEM (1:1) (Gibco) supplemented with 20% FCS and penicillin (100 U/mL) streptomycin (100 μg/mL) at 37°C and 5% CO_2_. Cells were passaged when they reached 70-80% confluence at 1:5-6 with 0.05% trypsin. The shRNAmir pSM2 plasmids (OpenBiosystems, Madrid, Spain) have been described previously [4]. Cells were transfected for 3-4 h in OptiMEM (Invitrogen) using Lipofectamine Plus (Invitrogen). For western blots, 5 × 10^5 ^cells were transfected using 1.5 μg DNA (0.75 μg of each shRNA plasmid), 10 μL of Plus reagent and 5 μL of Lipofectamine. After 14-16 h, puromycin (1 μg/ml) was added and cells were grown for 3 d prior to lysis in radioimmunoprecipitation assay (RIPA) buffer. For immunofluorescence, 10^5 ^cells were plated on collagen-coated coverslips and transfected with 150 ng of each pSM2 plasmid plus 50 ng pEGFP (Clontech) using 2 μl Plus reagent and 1 μl Lipofectamine, cultured for 72 h and then fixed.

### Immunofluorescence

Cells were fixed in 4% paraformaldehyde (PFA) for 15 min and permeabilised with 0.1% Triton for 15 min at room temperature, nonspecific binding was blocked for at least 30 min using blocking buffer (PBS, 50 mM glycine, 2% bovine serum albumin (BSA), 0.01% sodium azide). Next, cells were incubated for 2 h with primary antibodies (Table 1) in wash buffer (blocking buffer diluted 1:10 in PBS), washed four times, 5 min each, and incubated with Cy3 or Cy5 anti-mouse IgG and Texas Red anti-rabbit IgG donkey antibodies diluted 1:500 in wash buffer. After washing again, the coverslips were mounted using Vectashield^® ^(Vector Labs, Burlingame) containing 4',6-diamidino-2-phenylindole (DAPI, 1.5 μg/mL), and wide-field epifluorescence images were acquired at room temperature on an upright fluorescence microscope (AxioimagerD1; Carl Zeiss, Inc.) using a 40 × 0.75 NA objective (Figures 2A and 3A) an a HRm camera and Axiovision 4.8 acquisition software. Confocal images (Figures 1, 2B-D and 3B-D), were acquired at room temperature on a DM IRE2 laser scan microscope (Leica) with a 63 × 1.4 NA objective, an optimised pinhole and x2 electronic zoom using LCS acquisition software. Images are presented after digital adjustment of curve levels (gamma) to maximize signal with ImageJ software. In all cases, exposure times and digital manipulation were the same for control and experimental samples. Fluorochromes and colours are as indicated in the figure legends.

**Table 1:** list of antibodies used

Antibody	Western	IF	*Source*
Anti- GSK-3α mouse monoclonal antibody	1:5000	1:500	Santa Cruz (sc-5264)
Anti- GSK-3β mouse monoclonal antibody	1:5000	1:500	BD Biosciences (610202)
Anti- phospho Ser^21^-GSK-3α rabbit monoclonal antibody	1:1000	1:100	Cell Signalling (9316)
Anti- phospho Ser^21/9^-GSK-3α/β rabbit polyclonal antibody	1:1000	1:100	Cell Signalling (9331)
Anti- phospho Tyr^216^-GSK-3β mouse monoclonal antibody	1:1000	1:100	BD Biosciences (612313)
Anti- phospho Tyr^279/216^-GSK-3α/β rabbit polyclonal antibody	1:1000	1:100	Abcam (ab52188)
Anti- phospho Tyr^279/216^-GSK-3α/β rabbit polyclonal antibody	1:1000	1:100	Merck (ST1013)
Anti- tubulin mouse monoclonal antibody	N.A.	1:100	Chemicon (CBL412)
Anti- Ki67 rabbit polyclonal antibody	N.A.	1:100	Abcam (ab833)

### Western blot analysis

Lysates were prepared by incubating cells on ice for 5 min in RIPA buffer [29], cleared by centrifugation, frozen and stored at -80°C and then thawed again at 4°C. Protein concentration was determined by the Lowry method and 20 μg per lane of lysate was resolved by 10% SDS-polyacrylamide gel electrophoresis (PAGE) and transferred onto Protran^® ^(Whatman) nitrocellulose membranes. Nonspecific binding was blocked by 1 hour incubation with blocking buffer before membranes were probed overnight at 4°C with primary antibodies diluted in blocking buffer (3% BSA in Tris-buffered saline with 0.1% Tween-20 (TBS-T)). After extensive washing with TBS-T, specific bands were detected on Hyperfilm™ (GE Healthcare) using horseradish peroxidase (HRP)-conjugated donkey secondary antibodies (1:10,000; Jackson Labs) and the ECL detection system (GE Healthcare). Primary antibodies are listed in Table I.

## Competing interests

The authors declare that they have no competing interests.

## Authors' contributions

VMC conducted and analysed the data and drafted the manuscript; RK analysed the data and drafted the manuscript.

## Supplementary Material

Additional file 1 **Supplemental Figure 1. Coexpression of GFP and shRNA plasmids**. 22Rv1 cells were transfected with a combination of the αsh2, βsh2 and eGFP plasmids. 72 hours after transfection, cells were fixed and stained with anti-GSK-3α or anti-GSK-3β monoclonal antibodies (red) and analyzed by fluorescence microscopy. Scale bars = 100 μm. Click here for file

Additional file 2 **Supplemental Video. Z-series of images to visualise nonspecific antigens detected by anti-PTyr GSK-3 antibodies**. Cells transfected with eGFP, αsh2 and βsh2 plasmids were fixed and stained using anti-GSK-3α + anti-GSK-3β monoclonal antibodies (red) and anti-GSK-3α/β pY279/216 from Merk (far red, shown as white in GFP transfected cells). Images were obtained using a confocal microscope as described in the Methods. The video shows a Z-stack of 5 optical sections from bottom (surface contact with coverslip) to top. Click here for file
