# Supplementary material for: Issues associated with the use of phosphospecific antibodies to localise active and inactive pools of GSK-3 in cells
Source: Biol Direct. 2011 Jan 24;6:4. doi: 10.1186/1745-6150-6-4 (PMC3039639; doi:10.1186/1745-6150-6-4)
Supplement: Additional file 1 — Supplemental Figure 1. Coexpression of GFP and shRNA plasmids. 22Rv1 cells were transfected with a combination of the αsh2, βsh2 and eGFP plasmids. 72 hours after transfection, cells were fixed and stained with anti-GSK-3α or anti-GSK-3β monoclonal antibodies (red) and analyzed by fluorescence microscopy. Scale bars = 100 μm. [file 1745-6150-6-4-S1.DOC]

shα46 + shβ92 + eGFP

GSK-3α

GSK-3β

Merge

Merge

GFP

GFP
